# Temporal and socioeconomic trends in hospital admissions for miscarriage and ectopic pregnancy in England, 2004–2024: a nationwide study

**DOI:** 10.1016/j.lanepe.2026.101777

**Published:** 2026-07-29

**Authors:** Sindhu Sekar, Joshua Odendaal, Siobhan Quenby, Nicola Tempest

**Affiliations:** a Department of Women's and Children's Health, Centre for Women's Health Research, Institute of Life Course and Medical Sciences, University of Liverpool, Liverpool Health Partners, Liverpool, L8 7SS, UK; b Hewitt Centre for Reproductive Medicine, Liverpool Women's NHS Foundation Trust, Liverpool, L8 7SS, UK; c Liverpool Women's NHS Foundation Trust, Liverpool Health Partners, Liverpool, L8 7SS, UK; d NIHR Applied Research Collaboration Northwest Coast, University of Liverpool, Liverpool, UK; e Tommy's National Centre for Miscarriage Research, University Hospitals Coventry and Warwickshire NHS Trust, Coventry, CV2 2DX, UK; f Warwick Medical School, University of Warwick, Coventry, CV4 7AL, UK

**Keywords:** Joinpoint, Miscarriage, Ectopic pregnancy, Early pregnancy, Deprivation

## Abstract

**Background:**

Miscarriage and ectopic pregnancy are among the most common early pregnancy complications, yet contemporary national trends and associated inequalities in England remain poorly characterised. This study examined 20-year temporal patterns in hospital admissions for miscarriage and ectopic pregnancy, with additional stratification by socioeconomic deprivation and age.

**Methods:**

National admissions data from 2004 to 2024, curated by NHS England, were extracted from Hospital Episode Statistics and the Maternity Services Data Set. Annual counts of miscarriage (ICD-10 O02–O03) and ectopic pregnancy (ICD-10 O00), deliveries, deprivation deciles, and age groups were analysed. Joinpoint regression (version 5.0) was used to identify significant changes in trends, calculating Annual Percent Change (APC) and Average Annual Percent Change (AAPC).

**Findings:**

Miscarriage admissions declined significantly between 2018 and 2021 (APC = −4.44∗%; 95% CI: −7.4218 to −0.7135; *p* = 0.03), followed by a modest rise after 2021 (APC = +2.823%; 95% CI: −1.8311 to 8.8970; *p* = 0.13). Ectopic pregnancy admissions increased significantly up to 2012 (APC +2.82%; 95% CI: 1.3845–3.8309; *p* = 0.007), declined with the introduction of conservative management guidelines (APC = −2.97%; 95% CI: −3.9393 to 3.827; *p* = 0.15) and rose again after 2021 (APC = +4.23∗% (95% CI: 2.1194–7.1943; *p* < 0.001). A persistent deprivation gradient was observed, with the most deprived deciles consistently experiencing the highest admissions (around two-fold difference). The proportion of admissions among women aged 25–34 years increased (approximately 40–50%) for both outcomes.

**Interpretation:**

Persistent socioeconomic inequalities reflect a combination of population structure, higher prevalence of modifiable risk factors and inequitable access to care. Strengthening equitable access, tailored outreach through digital platforms and preventive strategies addressing modifiable risk factors may help optimise early pregnancy care and reduce avoidable emergency admissions. Findings should be interpreted with caution due to limited risk factor, parity, and age–deprivation data.

**Funding:**

None.

**Research in context**

**Evidence before this study**

Before undertaking this study, we reviewed evidence on temporal trends in miscarriage and ectopic pregnancy using searches of MEDLINE from 1st January 1990 to 31st December 2023. Search terms included miscarriage, ectopic pregnancy, early pregnancy loss, hospital admissions, incidence, trends, Joinpoint, inequalities, and deprivation. We included studies reporting temporal trends in miscarriage and ectopic pregnancy and restricted inclusion to English language. Studies addressing termination of pregnancy admissions were excluded. Existing evidence primarily focused on clinical management, short-term outcomes, or regional cohorts, with limited national analyses. Few studies examined long-term temporal trends, and even fewer assessed socioeconomic inequalities or formally identified changes in trends over time. No previous study used Joinpoint regression to evaluate national trends in miscarriage and ectopic pregnancy admissions in England over two decades.

**Added value of this study**

This study provides the first national, 20-year Joinpoint regression analysis of miscarriage and ectopic pregnancy hospital admissions in England. By integrating temporal trends with socioeconomic deprivation and maternal age, it identifies statistically significant changes in admission patterns and demonstrates persistent deprivation-related inequalities that have not diminished over time. The findings link observed trend changes to evolving clinical guidelines, service reconfiguration (strengthening of early pregnancy units, greater reliance on remote consultation), and demographic shifts (advancing maternal age, declining fertility rate, delayed childbearing), offering insights beyond descriptive trend analyses.

**Implications of all the available evidence**

Together with existing evidence, these findings indicate that increase in admissions after 2020 is likely to be multifactorial and may reflect a combination of service configuration (changes in referral thresholds, healthcare-seeking behaviour, or more cautious clinical management following the pandemic), population demographics (accrual of risk factors such as obesity, smoking, endometriosis, pelvic inflammatory disease) and broader health factors (combined effect of evolving maternal risk profiles). Persistent socioeconomic disparities likely reflect a combination of population structure, with women of reproductive age more concentrated in deprived areas, higher prevalence of modifiable risk factors, and inequitable access to care. This highlights the need for intensifying equitable access to early pregnancy services, workforce capacity, tailored outreach through digital platforms (e.g. podcasts and social media) and further research into modifiable risk factors underlying these inequalities. Long-term surveillance integrating individual-level risk factors and patient-reported outcomes is essential to inform policy, service planning, and prevention strategies.

## Introduction

Early pregnancy complications, particularly miscarriage and ectopic pregnancy, represent a major clinical and public health concern.^1^ Miscarriage is defined as pregnancy loss before viability.^1^ Each year, it is estimated that approximately 250,000 miscarriages occur in the UK, affecting around one in five pregnancies.^2^ Globally, an estimated 23 million pregnancy losses occur each year,^3^ making miscarriage and other forms of early pregnancy loss a major contributor to maternal morbidity and mortality. These events impose considerable physical, psychological, and social burdens on women of reproductive age and represent a significant yet often under-recognised component of global maternal health.^1^

Alongside this, there are approximately 11,000 hospital admissions for ectopic pregnancy each year in the UK.^4^ Ectopic pregnancy, defined as a pregnancy that implants outside of the uterine cavity, remains a major cause of early pregnancy morbidity.^4^

Pregnancy loss has been increasingly recognised as a marker of long-term health risk extending beyond pregnancy.^3^ Emerging evidence links recurrent pregnancy loss with an elevated risk of cardiovascular disease and venous thromboembolism.^3^ While the physical consequences of miscarriage are substantial, the psychological impact warrants equal attention. Miscarriage can precipitate significant grief, trauma, and anxiety, yet these experiences often go unrecognised by healthcare professionals, families, and society.^3^

From a health systems perspective, economic analyses demonstrate considerable variation in the direct costs of miscarriage management across countries and care settings. Reported direct costs for expectant management range from approximately £380 in the United States of America (USA)^3^ to £1067 in Hong Kong,^3^ whereas medical management costs vary between £298 in the USA and £1421 in the UK.^3^ Early pregnancy loss contributes to a substantial burden on healthcare services, accounting for over 50,000 hospital admissions annually.^2^

Despite their clinical and public health significance, national trend data on miscarriage and ectopic pregnancy remain scarce, particularly analyses that examine long-term temporal changes in England.

Time trend analyses are widely used by government agencies and public health researchers to describe and monitor patterns of change in key outcomes related to environmental, economic, and health issues.^5^ A variety of statistical approaches can be applied to identify points in time where trends change in rate or direction, including pairwise tests, trend contrasts, and parametric regression models such as linear, logistic, or multinomial regression.^5^

Among these approaches, Joinpoint regression analysis, used by the National Cancer Institute (NCI), has become one of the most robust and widely used methods for evaluating temporal trends.^5^ Originally designed to analyse cancer incidence and mortality rates, this technique has since been widely adopted in public health research for evaluating temporal trends in a variety of health outcomes.^6^

We aimed to use Joinpoint regression analysis to examine twenty-year trends in common complications of early pregnancy such as miscarriage and ectopic pregnancy admissions in England (2004–2024) and to identify periods where statistically significant changes in trends occurred. Understanding long-term trends in miscarriage and ectopic pregnancy is essential to improve the quality, equity, and efficiency of early pregnancy care in England. Despite their frequency and profound personal impact, these conditions remain under-characterised at a population level, particularly with respect to socioeconomic inequalities, maternal age, and changing patterns of hospital utilisation. Analysing national surveillance data over time enables us to detect significant inflection points in national trends that may correspond to policy or service changes, identify areas of persistent inequality to guide targeted public health interventions and equitable resource allocation, and generate evidence to support service improvement.

## Methods

### Data sources

We sourced publicly available data on miscarriage and ectopic pregnancy from the Hospital Episode Statistics (HES) and Maternity Services Data Set (MSDS) curated by NHS England. HES is a national administrative database containing detailed records of all hospital admissions, outpatient appointments, and historical accident and emergency attendances at NHS hospitals in England.

The Maternity Services Data Set (MSDS) collects information across each stage of the maternity care pathway in NHS-funded maternity services and captures additional clinical and service-level information not available in HES. In this study, HES and MSDS were used for distinct and complementary purposes and were not linked at the individual level. HES was used to identify hospital admissions for miscarriage and ectopic pregnancy, while MSDS was used to derive maternity-related denominators (deliveries). As these datasets capture different components of care pathways, the risk of double counting was minimised. NHS England applies standardised XML schema validation, data cleaning, and quality assurance processes, including internal consistency checks (e.g. a single booking per woman per reporting period per organisation) to minimise double counting. Full methodological details are described in the Supplementary Folder—Methods.docx.

Annual counts of deliveries, miscarriages (ICD-10 codes O02. 0–O02.9, O03), and ectopic pregnancies (ICD-10 O00) were extracted for the period 2004–2024. Data were stratified by Index of Multiple Deprivation (IMD) deciles to assess socioeconomic gradients (Decile 1 as most deprived and Decile 10 as least deprived) and by maternal age groups to examine age-related distributions for the period 2013–2024.

We attempted to extract data stratified jointly by age and IMD in relation to pregnancy outcomes; however, publicly available MSDS/HES data provide counts stratified by age and deprivation decile separately, without cross-tabulated data. Analyses were therefore performed independently for age and deprivation. Individual-level record linkage was not available, precluding assessment of their independent effects.

All extracted datasets were collated using Microsoft Excel (Microsoft Corporation, Redmond, WA, USA) before importing summary tables into the Joinpoint Regression Program (version 5.0, National Cancer Institute, USA) for trend analysis.

### Statistical analysis

We analysed temporal trends in miscarriage and ectopic pregnancy admissions using the Joinpoint Regression Program (version 5.0; National Cancer Institute, USA). Joinpoint regression identifies statistically significant changes in temporal trends by fitting a series of straight lines on a logarithmic scale, with each “Joinpoint” representing a significant change in slope.

A log-linear model with Poisson variance was applied to annual counts of miscarriage and ectopic pregnancy using deliveries as the proxy denominator. Model selection was guided by the Weighted Bayesian Information Criterion (BIC), and the Monte Carlo permutation test was used to determine the optimal number of Joinpoint.

The Annual Percent Change (APC) quantifies the yearly change in rates, assuming a constant percentage change per year. The Average Annual Percent Change (AAPC) provides a single summary measure of the trend over a fixed interval (2004–2024), calculated as a weighted average of the APCs from each segment, with weights proportional to the segment lengths.

For trend directions, “significant increase/decrease” meant a statistically significant trend slope (*p* < 0.05, based on AAPC). Non-significant trends (*p* > 0.05) were labelled “non-statistically significant increase/decrease” or “stable” based on AAPC values. Statistical significance was defined as *p* < 0.05.

Estimates are based on observed hospital admissions rather than modelled population incidence. This study complies with the GATHER recommendation (Supplementary File).

### Role of funding source

There was no funding source for this study.

### Ethics and informed consent

This study involved the analysis of Hospital Episode Statistics and the Maternity Services Data Set and did not involve any collection or use of human data. Therefore, ethical approval was not required for this study.

## Results

### Miscarriage

#### Admission trends

**Fig. 1:**
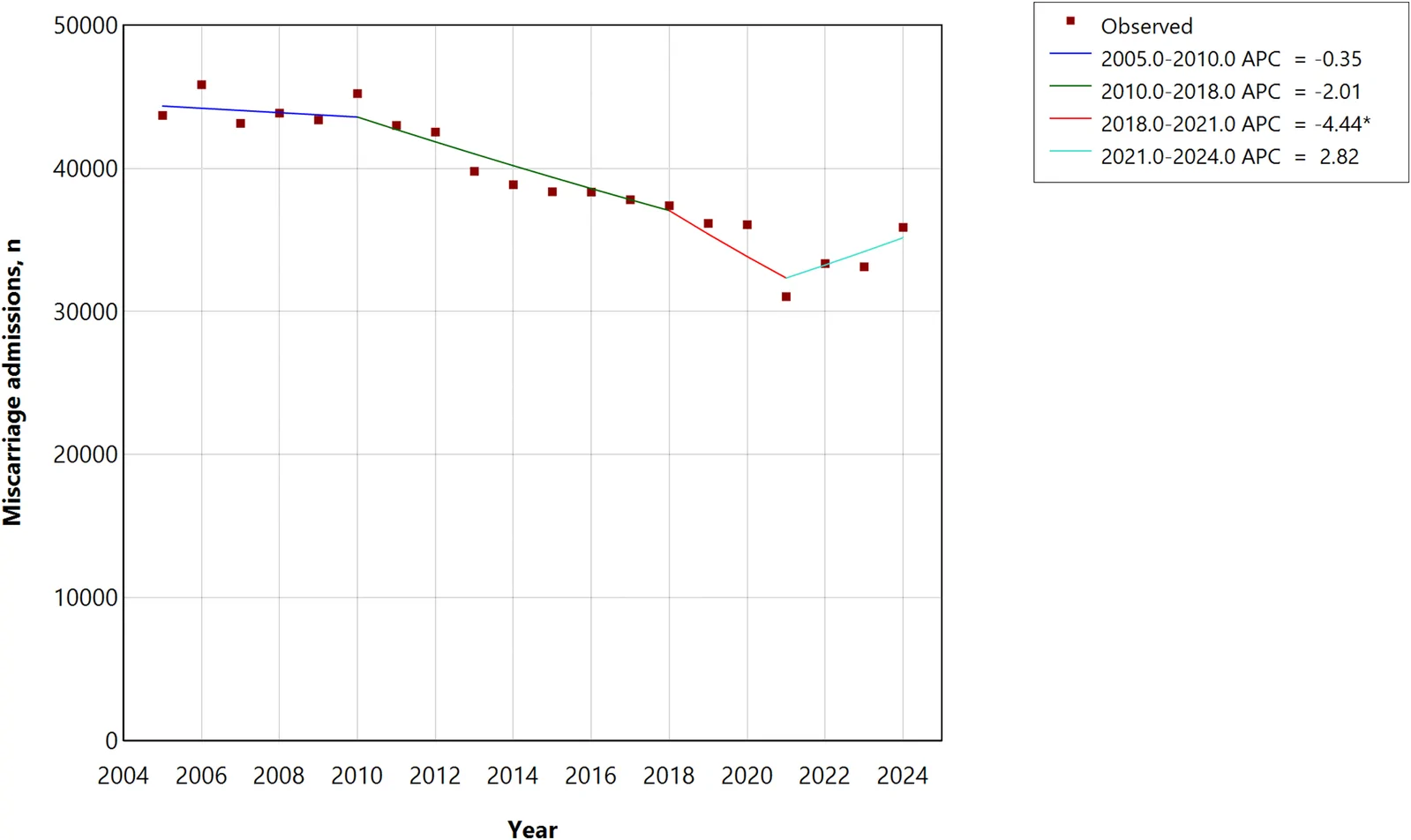
**Temporal trends in Miscarriage admissions (per 100,000 deliveries) in England, 2004**–**2024.** ∗Indicates that APC is significantly different from zero at the alpha = 0.05 level.

Between 2004 and 2024, the Joinpoint regression model identified three join points (2010, 2018, and 2021) in the trend of miscarriage admissions in England, indicating statistically significant changes in direction over time (Fig. 1 and Supplementary File–Table S1).

From 2004 to 2010, miscarriage admissions showed a slight, non-significant decline with an APC of −0.35 (95% CI: −4.7980 to 5.3489; *p* = 0.77). Between 2010 and 2018, a non-significant decrease was observed (APC = −2.01%; 95% CI: −5.1570 to −2.9693; *p* = 0.30), representing a non-significant downward trend.

In contrast, from 2018 to 2021, the trend showed a significant decrease (APC = −4.44∗ %; 95% CI: −7.4218 to −0.7135; *p* = 0.03), followed by a continued but non-significant rise from 2021 to 2024 (APC = +2.823%; 95% CI: −1.8311 to 8.8970; *p* = 0.13).

Overall, miscarriage admissions declined significantly during 2018-2021, with subsequent years showing a modest upturn that did not reach statistical significance.

#### Deprivation

**Fig. 2:**
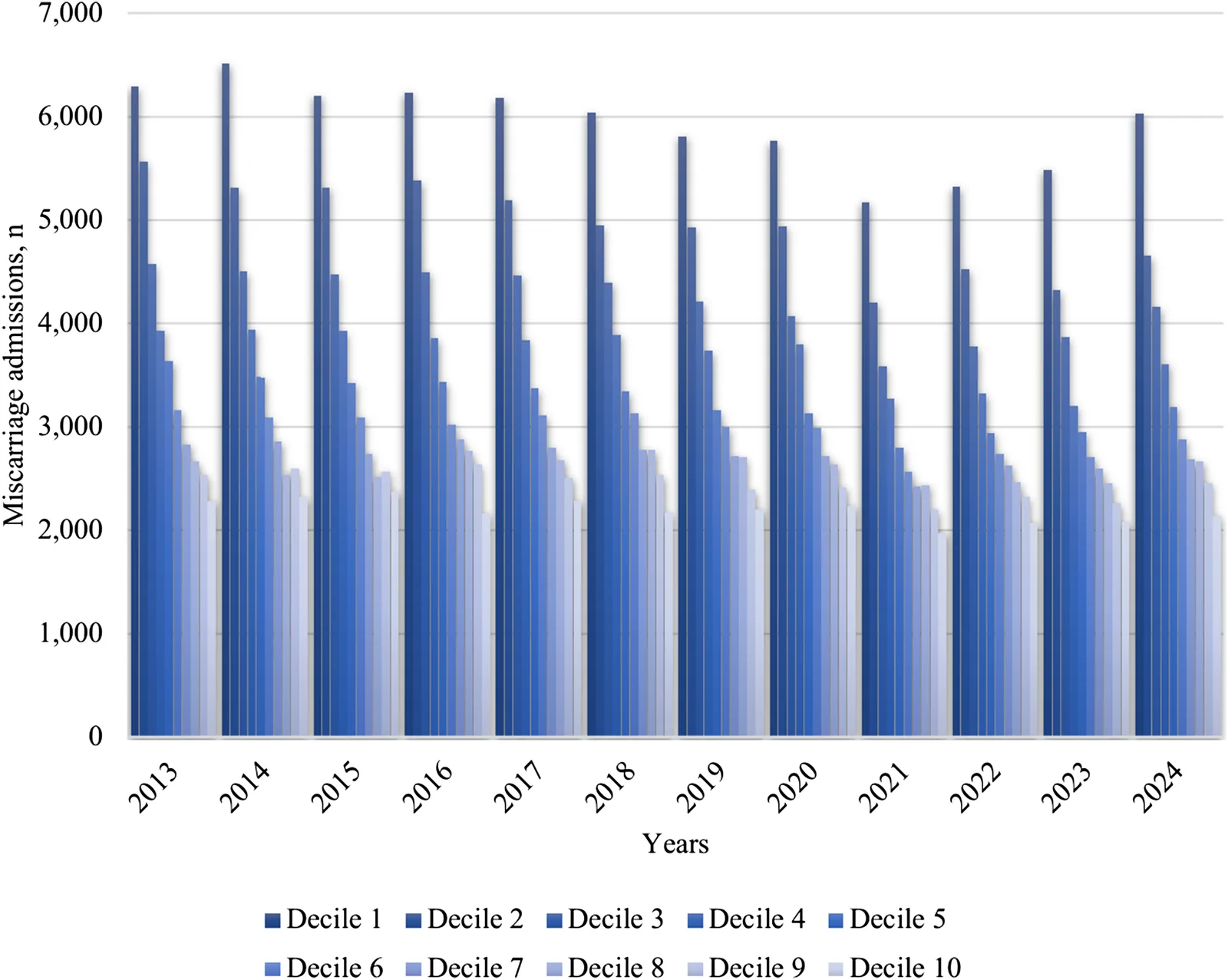
**Distribution of miscarriage admissions by deprivation decile in England, 2013**–**2024. (Decile 1- Most deprived)**.

During 2013–2024, miscarriage admissions demonstrated a pronounced and persistent socioeconomic gradient across deprivation deciles in England (Fig. 2). The most deprived deciles (1–3) consistently accounted for the highest proportion of admissions each year, whereas the least deprived deciles (8–10) contributed the lowest. In 2024, the number of admissions in the most deprived decile was more than twice that observed in the least deprived decile.

This enduring pattern highlights marked inequalities in early pregnancy outcomes, with women living in the most deprived areas experiencing a disproportionate burden of miscarriage-related hospital activity. The stability of this gradient over time suggests that socioeconomic inequalities have not narrowed, despite national efforts to improve maternity safety and access to early pregnancy services.

#### Age distribution

During 2013–2024, most miscarriage admissions occurred among women aged 25–34 years, who accounted for approximately 40–50% of all cases annually (Supplementary File–Fig. S1). The proportion of miscarriages in the 35–39 years age group ranged from 20% to 25%, showing a gradual increase over the study period. Admissions among women aged 20–24 years declined slightly, representing 15–20% of cases each year, while those aged under 20 years consistently contributed less than 5% of admissions.

Miscarriages among women aged 40 years and older comprised a smaller but gradually rising proportion, increasing from around 8% in 2013 to nearly 12% in 2024.

### Ectopic pregnancy

#### Admission trends

**Fig. 3:**
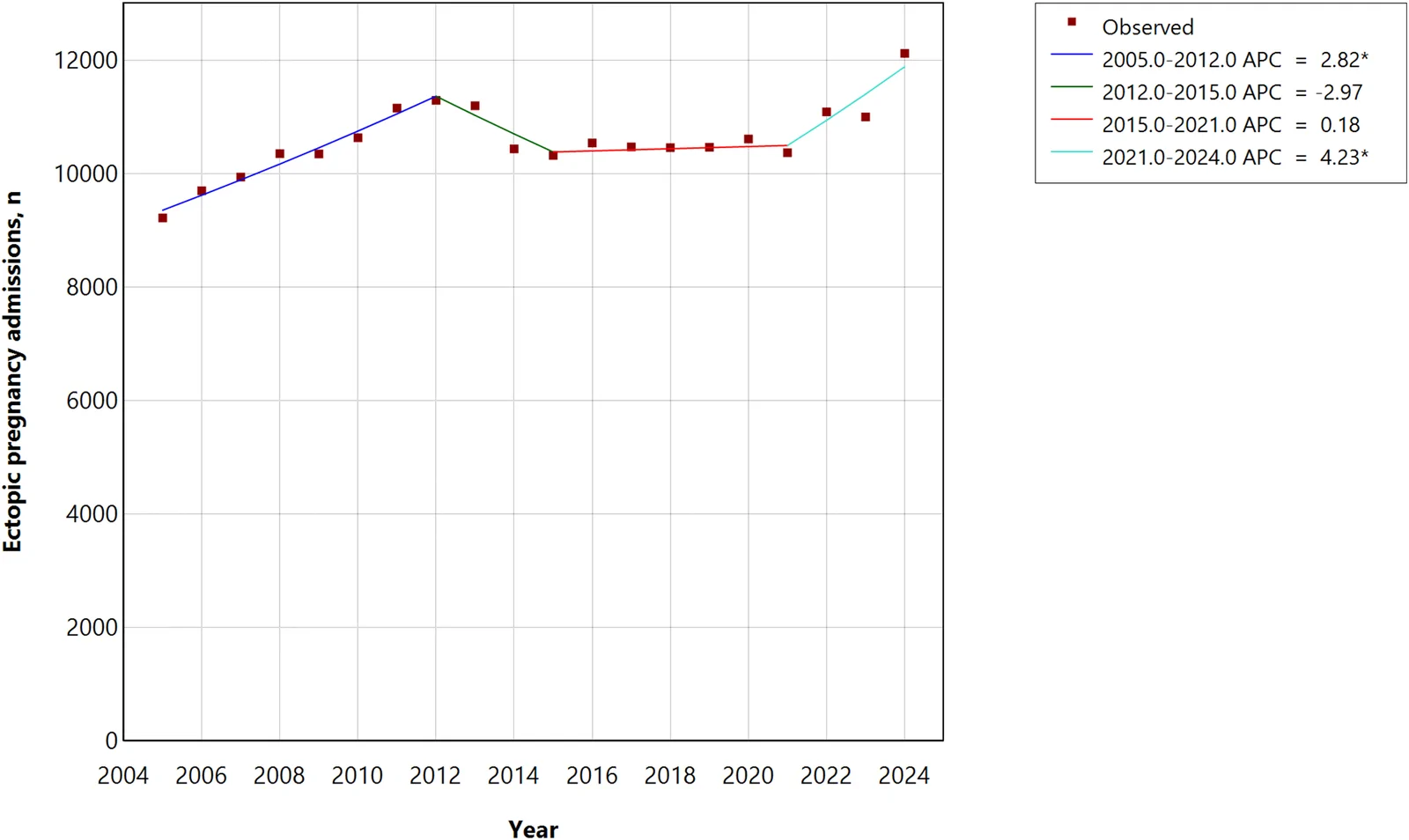
**Temporal trends in ectopic pregnancy admissions (per 100,000 deliveries) in England, 2004**–**2024.** ∗Indicates that APC is significantly different from zero at the alpha = 0.05 level.

From 2004 to 2024, the Joinpoint regression model identified three join points (2012, 2015, and 2021) in the trend of ectopic pregnancy admissions in England (Fig. 3 and Supplementary File–Table S2). This indicates four distinct temporal segments with periods of both increase and decline.

Between 2004 and 2012, ectopic pregnancy admissions showed a significant increase, with an APC of +2.82∗% (95% CI: 1.3845–3.8309; *p* = 0.007). This was followed by a non-significant decline from 2012 to 2015 (APC = −2.97%; 95% CI: −3.9393 to 3.827; *p* = 0.15) and a period of stability between 2015 and 2021 (APC = +0.18%; 95% CI: −1.6374 to 1.5835; *p* = 0.66).

In the most recent segment (2021–2024), the model detected a significant upward trend, with an APC of +4.23∗% (95% CI: 2.1194–7.1943; *p* < 0.001), representing the highest rate of increase across the study period.

Overall, ectopic pregnancy admissions demonstrated an initial rise to 2012, a period of relative stability between 2012 and 2021, and a renewed increase after 2021.

#### Deprivation

**Fig. 4:**
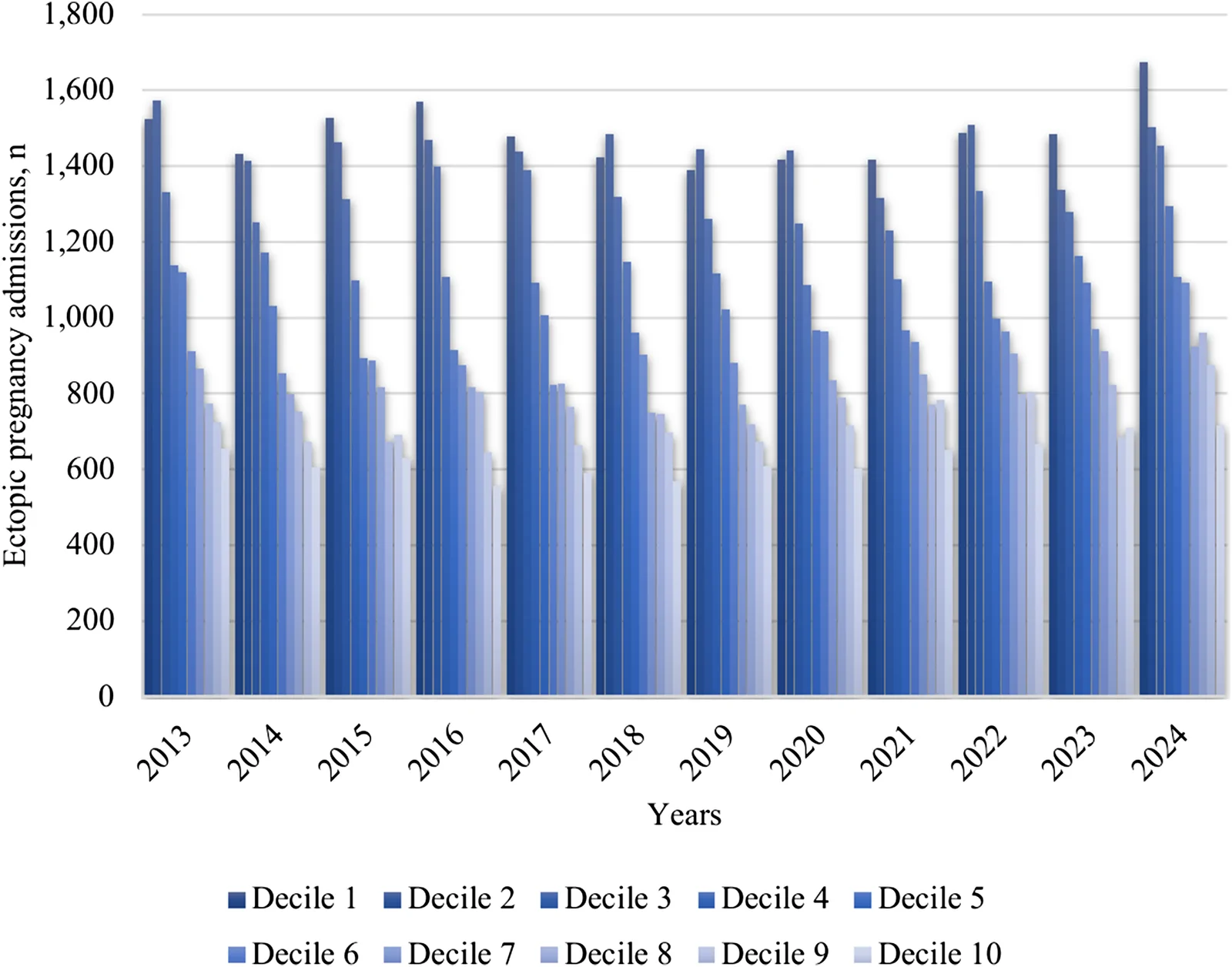
**Distribution of Ectopic pregnancy admissions by deprivation decile in England, 2013–2024. (Decile 1- Most deprived)**.

During 2013–2024, ectopic pregnancy admissions showed a clear and persistent socioeconomic gradient across deprivation deciles in England (Fig. 4). The most deprived deciles (1–3) consistently accounted for the largest share of admissions each year, whereas the least deprived deciles (8–10) had the lowest. In 2024, the number of admissions recorded in the most deprived decile was more than double that in the least deprived decile, reflecting a strong deprivation gradient that remained stable throughout the study period.

These findings highlight enduring inequalities in reproductive health outcomes, with women from more deprived communities disproportionately affected by ectopic pregnancy.

#### Age distribution

During 2013–2024, most ectopic pregnancy admissions occurred among women aged 25–34 years, accounting for approximately 45–55% of cases annually (Supplementary File–Fig. S2). The 35–39 years age group contributed a further 20–25%, while admissions among women aged 20–24 years remained stable at around 15–18% each year. The proportion of cases among women aged under 20 years was consistently low, representing less than 5% of total admissions throughout the period.

Notably, the proportion of ectopic pregnancies among women aged 40 years and older showed a gradual increase from around 7% in 2013 to nearly 12% in 2024, indicating a rising trend in advanced maternal age at conception.

## Discussion

Our study provides the first national-level analysis of temporal and socioeconomic trends in miscarriage and ectopic pregnancy admissions in England over a 20-year period (2004–2024) using Joinpoint regression. We observed a significant decline in miscarriage admissions between 2010 and 2018, followed by a modest upward trend after 2018, indicating a reversal of the long-term downward trajectory. In contrast, ectopic pregnancy admissions demonstrated a gradual increase, with a significant rise after 2021.

Across both conditions, a persistent deprivation gradient was evident, women living in the most deprived deciles consistently experienced higher rates of hospital admissions than those in the least deprived areas. This inequality remained stable over time, suggesting that improvements in maternity and reproductive health outcomes have not been equally distributed across socioeconomic groups.

Analysing temporal trends using national surveillance data presents several challenges, these include changes in diagnostic coding and clinical terminologies to align with evolving clinical practice, variability in reporting and administrative processes across National health service (NHS) trusts, progressive improvements in data quality and coverage over time. Such factors may introduce artefactual fluctuations in observed trends and should be considered when interpreting long-term changes in miscarriage and ectopic pregnancy admissions.

The establishment of Early Pregnancy Assessment Units (EPAUs) in UK hospitals represented a major advance in the management of early pregnancy complications,^7^ enabling prompt diagnosis, outpatient management, and reduction in unnecessary hospital admissions.^7^ Prior to the introduction of EPAUs, surgical evacuation of retained products of conception was the primary method of miscarriage management, as outlined in the 2006 Royal College of Obstetricians and Gynaecologists (RCOG) Green-top Guideline.^8^

The subsequent 2012 National Institute for Health and Care Excellence (NICE) guideline marked a paradigm shift by advocating conservative and medical management as safe and effective alternatives to surgery.^9^ This evolution in clinical guidance, together with the widespread implementation of EPAUs, likely contributed to the observed reduction in miscarriage-related hospital admissions during the 2010–2018 period, reflecting a shift from inpatient surgical management to outpatient and expectant care pathways.

The resurgence of miscarriage admissions after 2020 observed in our analysis, though moderate, warrants further exploration. Studies have examined the potential association between COVID-19 infection, Covid 19 vaccination and the risk of miscarriage,^10^^,^^11^ Systematic reviews of clinical data largely do not support a causal link between vaccination and miscarriage.^10^ However, immunopathological studies have proposed plausible biological mechanisms through which viral infection and the associated inflammatory response might increase the risk of early pregnancy loss.^11^

Beyond infection-related factors, several population-level risk determinants may have contributed to the post-2020 rise in miscarriage admissions. These include the progressive increase in maternal age at conception in the UK,^12^ rising prevalence of obesity,^12^ and broader environmental and lifestyle changes^12^ all of which are independently associated with miscarriage risk. The restructuring of EPAUs during the COVID-19 pandemic,^13^ with an increased emphasis on outpatient and remote care,^13^ may also have influenced admission patterns. Qualitative studies during this period have highlighted the emotional and psychological strain experienced by women navigating early pregnancy complications in the context of limited in-person support.^14^ Increased admissions may partly reflect rising medicolegal pressures and defensive practice during the COVID-19 pandemic, promoting more cautious, intervention-led care.^15^ Mifepristone is now recommended by NICE, in combination with misoprostol, for the management of missed miscarriage and improves treatment success. As a recent change in practice, its effects on admission patterns and care pathways are likely to emerge in the coming years.^16^ These service adaptations, together with evolving risk profiles, may collectively explain the rebound increase in miscarriage-related hospital admissions in the years following the pandemic.

The rise in ectopic pregnancy admissions between 2004 and 2012, observed in this study, likely reflects contemporaneous diagnostic and management practices. During this period, national protocols recommended surgical management, principally laparoscopy or laparotomy, as the standard of care for confirmed ectopic pregnancy.^17^ The subsequent decline in admissions between 2012 and 2015 coincides with the publication of updated NICE guidelines promoting medical and conservative management,^9^ supported by growing evidence for the effectiveness and safety of methotrexate in selected cases.^9^ These shifts in practice likely contributed to reduced inpatient admissions for ectopic pregnancy.

However, the renewed increase in ectopic admissions in recent years, despite these guidelines, may be multifactorial. Contributing factors include the rising prevalence of recognised risk factors such as sexually transmitted infections,^4^^,^^12^ pelvic inflammatory disease,^4^^,^^12^ prior caesarean section,^4^^,^^18^ endometriosis,^4^^,^^19^ and the increasing use of assisted reproductive technologies.^4^^,^^20^ Advances in diagnostic sensitivity, particularly with high-sensitivity urine pregnancy tests capable of detecting pregnancy before a missed period,^21^ have also led to earlier ultrasound evaluation and a higher frequency of pregnancies of unknown location (PUL) being investigated or admitted for monitoring.

In addition, the COVID-19 pandemic and associated restructuring of EPAU^13^ may have contributed to a temporary rebound in surgical management.^14^

For ectopic pregnancy, admissions among women aged ≥40 years increased from around 7% in 2013 to 12% in 2024, consistent with the national shift toward delayed childbearing and potentially contributing to the recent rise in ectopic pregnancy incidence.^4^^,^^12^

The increase in miscarriage and ectopic pregnancy admissions among the most deprived populations underscores persistent socioeconomic inequalities in early pregnancy outcomes and highlights an urgent research priority to investigate the underlying causes, including differences in health behaviours, access to care, and broader social determinants of reproductive health. Age distribution varied by deprivation, with younger women more concentrated in deprived areas (20–25 years: 28·8% most deprived vs 10·6% least deprived; <20 years: 11·4% vs 2·2%),^22^ while older age groups were more prevalent in less deprived populations (30–35 years: 39·1% least deprived vs 20·2% most deprived).^22^ These patterns suggest that part of the observed socioeconomic gradient may reflect underlying population structure, with women in peak reproductive age groups more concentrated in deprived areas. Importantly, more deprived populations have a higher prevalence of key risk factors associated with pregnancy loss, including smoking,^12^ obesity,^23^ and sexually transmitted infections.^12^ For example, smoking prevalence is substantially higher in the most deprived areas (19%) compared with the least deprived (6%),^12^ and obesity affects 36% of adults in the most deprived quintile vs 22% in the least.^23^ In addition, access to early maternity care is lower in more deprived populations (56·9% vs 69·6%),^24^ potentially leading to delayed diagnosis and increased likelihood of hospital-based care.^24^ These findings suggest that the observed inequalities reflect a combination of population structure, differential exposure to risk factors, and inequitable access to care, rather than deprivation acting as a single causal factor. Although government strategies are in place to address these disparities such as improving access to healthy options (e.g. safe drinking water in public spaces) and strengthening preventive care through the healthcare workforce, their implementation and targeted education require intensification. Between 2004 and 2024, the characteristics of women giving birth in England and Wales have changed. The mean maternal age increased steadily from 29.0 years in 2004 to 31.0 years in 2024, indicating a shift towards delayed childbearing.^12^ Over the same period, the total fertility rate initially increased, peaking at 1.94 children per woman in 2010, before declining consistently to 1.41 in 2024, representing a historic low.^12^ Notably, the period after 2014 is characterised by a combination of increasing maternal age and a marked reduction in fertility, suggesting a change in reproductive behaviour.^25^ In the context of increasing maternal age, declining fertility rates, and rising childlessness, pregnancy loss may have broader implications beyond immediate clinical outcomes. The evidence suggests that pregnancy loss can influence subsequent fertility intentions and reproductive behaviour, particularly in the years following the event.^26^ Younger women may view pregnancy loss as a temporary interruption in their pathway to motherhood or progression to another child,^34^ potentially strengthening their desire to conceive. In contrast, for older women, pregnancy loss may more often signal the conclusion of their reproductive life.^26^ Additionally, the increasing prevalence of risk factors such as delayed childbearing, obesity, and other health conditions may contribute both to higher rates of pregnancy loss and to changes in fertility patterns.

This study provides a comprehensive national analysis of miscarriage and ectopic pregnancy admissions over two decades in England, using robust surveillance data and advanced statistical modelling. By applying Joinpoint regression, we were able to identify statistically significant changes in temporal trends and quantify the rate of change within each period. The inclusion of stratified analyses by deprivation and maternal age offers additional insight into the socioeconomic and demographic inequalities underlying early pregnancy outcomes. The use of nationally curated datasets (HES and MSDS) ensures wide population coverage and consistency with established NHS reporting systems.

However, several limitations should be acknowledged. First, this analysis relied on administrative data, which are subject to coding variation, changes in clinical terminology, and differences in reporting and clinical practice, admission thresholds, and service configuration across institutions may contribute to heterogeneity in observed trends. Such factors may introduce artefactual changes in temporal trends, particularly around major updates in coding or dataset structure. Second, data represent hospital admissions rather than true incidence and therefore may underestimate cases managed in community or outpatient settings, especially following the expansion of EPAUs and wider adoption of expectant management. Third, information on individual-level risk factors (e.g. smoking, BMI, reproductive history, and infection status) was not available, limiting adjustment for confounding variables. Importantly, parity data were not consistently available in the aggregated datasets and could not be incorporated**,** limiting assessment of its contribution to observed trends. Parity is closely linked to both fertility behaviour and pregnancy outcomes, and analyses incorporating birth order can provide additional insight into how reproductive patterns respond to demographic, economic, and policy changes over time.^27^ Evidence on the association between parity and miscarriage is mixed. A population-based study from Israel reported increasing miscarriage rates with higher parity^27^ and a higher proportion of registry-identified miscarriages among nulliparous women over time,^28^ whereas a US study found no clear association between parity and miscarriage incidence.^29^ In the absence of parity data, we were unable to distinguish between first and subsequent pregnancies, which may have different risk profiles and healthcare utilisation patterns. This limits our ability to determine whether the observed temporal trends reflect changes in population structure (e.g. delayed childbearing, declining fertility, or shifts in birth order distribution) or underlying clinical risk. In addition, Age and socioeconomic deprivation are likely to be correlated, and joint stratification would be ideal. However, publicly available MSDS/HES data provide counts stratified by age and deprivation separately, without cross-tabulation. As a result, their independent effects cannot be fully disentangled. Individual-level linked data are needed to examine their interaction. Fourth, although overdispersion was present in the aggregated data, Joinpoint regression (Poisson-based) was used as the standard method for estimating temporal trends (APC/AAPC).^30^ Negative binomial models are not supported within this framework; therefore, confidence intervals may be slightly underestimated, and results should be interpreted accordingly. Finally, IMD is a reasonable proxy for socioeconomic status; however, as an area-level measure, it does not fully capture individual-level deprivation and improvements in data quality, completeness, and coverage over time could partly explain some of the observed variations, and findings should therefore be interpreted with caution.

Despite these limitations, this study provides the most up-to-date and detailed assessment of temporal, socioeconomic, and demographic trends in miscarriage and ectopic pregnancy in England. The observed changes in admission patterns align with NHS priorities, including reducing avoidable hospital admissions and shifting towards preventive care.

Long-term trends in pregnancy loss are not characterised within national policy frameworks unlike other outcomes such as general hospital admissions.^31^ By providing a 20-year national analysis of trends this study addresses this evidence gap, bringing an under-recognised burden into focus and representing an initial step. The observed changes in trends are likely influenced by shifts in demographic characteristics and health service reconfiguration. The establishment of EPAUs^7^ and changes in service delivery during the COVID-19 period^13^ were associated with a marked reduction in admissions.^13^ In light of the persistent inequalities identified in our findings, we suggest that service provision should be aligned with population need, including strengthening EPAU capacity, integrating within care pathways, enhancing workforce training, and expanding access in underserved and deprived areas. National inequality reports (e.g. The King's Fund) demonstrate structural barriers driving higher reliance on emergency care in deprived populations,^32^ while our temporal analyses localise this pattern within pregnancy loss. For example–seven neighbourhoods in Blackpool rank among the ten most deprived in England,^33^ yet access to EPAU services in the area appears limited,^34^ highlighting a potential mismatch between population need and service provision. The persistence of socioeconomic gradients and evolving patterns of admission support the need for more targeted approaches, including education and outreach initiatives tailored to underserved groups similar to sugar tax.^35^ In particular, digital and social media-based interventions such as reels and podcasts may offer opportunities to engage populations less likely to access traditional health information channels. With widespread access to mobile phones and social media, digital platforms such as podcasts can deliver tailored, patient-centred health education to improve engagement in high-risk populations.^36^ Together, these findings support a shift towards access-driven early care models such as EPAUs, aligned with national priorities to optimise maternity care pathways, reduce emergency care burden, and address pressures on gynaecology waiting lists. Alongside this, we emphasise the need to strengthen targeted, equity-focused interventions and prevention strategies addressing modifiable risk factors. This includes policy measures such as sugar taxation, smoking cessation through tax, school-based health education, and wider use of digital and social media for public engagement. Service configuration should also be optimised, including coordinated referral pathways (e.g. centralised EPAU connect) to improve access and timely care. The limitations emphasise a need to strengthen data systems, including improved data completeness, linkage, and coverage. Enhanced data infrastructure, like approaches used in Scandinavian countries,^37^ would enable more precise monitoring of pregnancy loss and facilitate more effective, data-driven policy and service development.

## Contributors

S.S. contributed to conceptualisation, data access, data curation, formal analysis, verification of the underlying data, methodology, project administration, resources, software, validation, writing—original draft, and writing—review and editing.

S.S. had full access to all study data.

S.S., O.J., S.Q., and N.T. verified the underlying data.

O.J., S.Q., and N.T. contributed to data verification, supervision, validation, visualisation, project administration, and writing—review and editing.

S.S., O.J., S.Q., and N.T. were responsible for the decision to submit the manuscript for publication.

## Data sharing statement

Data collected for this study will be made available following publication. Any researcher may access the data for reasonable scientific purposes by contacting the corresponding author. No additional restrictions or formal data access agreements will apply.

## Declaration of interests

All authors declare no competing interests.
