# Supplementary material for: Temporal and socioeconomic trends in hospital admissions for miscarriage and ectopic pregnancy in England, 2004–2024: a nationwide study
Source: Lancet Reg Health Eur. 2026 Jul 29;68:101777. doi: 10.1016/j.lanepe.2026.101777 (PMC13559920; doi:10.1016/j.lanepe.2026.101777)
Supplement: Supplementary file 1 [file mmc1.docx]

**Supplementary file - Figure 1:** **Distribution of miscarriage admissions by maternal age group in England, 2013–2024***.*

Miscarriage admissions, n

| Years | <20 | 20-24 | 25-29 | 30-34 | 35-39 | 40-44 | 45-49 | > 50 |
| --- | --- | --- | --- | --- | --- | --- | --- | --- |
| 2013 | 1993 | 5842 | 8589 | 9809 | 7635 | 3845 | 366 | 609 |
| 2014 | 1869 | 5619 | 8731 | 9829 | 7540 | 3694 | 335 | 504 |
| 2015 | 1578 | 5358 | 8428 | 9781 | 7821 | 3588 | 336 | 21 |
| 2016 | 1510 | 5130 | 8620 | 9968 | 8030 | 3514 | 333 | 21 |
| 2017 | 1385 | 4759 | 8300 | 9983 | 8185 | 3694 | 385 | 26 |
| 2018 | 1322 | 4738 | 8223 | 9747 | 8343 | 3486 | 352 | 28 |
| 2019 | 1402 | 4275 | 7793 | 9810 | 8049 | 3550 | 346 | 14 |
| 2020 | 1252 | 4217 | 7722 | 9942 | 8086 | 3502 | 396 | 20 |
| 2021 | 875 | 3539 | 6613 | 8999 | 7165 | 3317 | 297 | 25 |
| 2022 | 981 | 3624 | 6837 | 9388 | 7723 | 3486 | 324 | 21 |
| 2023 | 1066 | 3558 | 6960 | 9333 | 7617 | 3396 | 324 | 28 |
| 2024 | 1017 | 3883 | 7453 | 10336 | 8298 | 3526 | 330 | 24 |

**Supplementary file - Figure 2. Distribution of ectopic pregnancy admissions by maternal age group in England, 2013–2024.**

Ectopic pregnancy admissions, n

| Years | < 20 | 20-24 | 25-29 | 30-34 | 35-39 | 40-44 | 45-49 | >50 |
| --- | --- | --- | --- | --- | --- | --- | --- | --- |
| 2013 | 346 | 1731 | 2981 | 3178 | 1876 | 591 | 37 | 6 |
| 2014 | 248 | 1525 | 2863 | 3033 | 1866 | 518 | 39 | 9 |
| 2015 | 260 | 1489 | 2888 | 3084 | 1858 | 494 | 52 | 2 |
| 2016 | 282 | 1377 | 2872 | 3159 | 2040 | 523 | 32 | 5 |
| 2017 | 228 | 1273 | 2826 | 3197 | 2115 | 505 | 33 | 2 |
| 2018 | 214 | 1309 | 2876 | 3159 | 2067 | 478 | 38 | 1 |
| 2019 | 205 | 1197 | 2774 | 3264 | 2074 | 500 | 33 | 1 |
| 2020 | 254 | 1244 | 2661 | 3306 | 2155 | 603 | 40 | 6 |
| 2021 | 198 | 1198 | 2556 | 3414 | 2195 | 565 | 32 | 9 |
| 2022 | 190 | 1244 | 2600 | 3668 | 2351 | 580 | 40 | 5 |
| 2023 | 236 | 1144 | 2776 | 3573 | 2318 | 547 | 39 | 13 |
| 2024 | 268 | 1255 | 2943 | 4049 | 2620 | 628 | 51 | 9 |

**Supplementary file –**

**Table 1**- **. Annual Percent Change (APC) in Miscarriage admissions(per 100,000 deliveries) in England, 2005–2024.**

| Estimated JoinPoint | | | | | | | |
| --- | --- | --- | --- | --- | --- | --- | --- |
| Joinpoint | | Estimate | | Lower CI | | Upper CI | |
| 1 | | 2010 | | 2007 | | 2015 | |
| 2 | | 2018 | | 2010 | | 2019 | |
| 3 | | 2021 | | 2018 | | 2022 | |
| Annual Percentage Change (APC) | | | | | | | |
| Segment | Lower Endpoint | Upper Endpoint | APC | Lower CI | Upper CI | Test Statistic | P-Value |
| 1 | 2004 | 2010 | -0.3487 | -4.7980 | 5.3489 | -- | 0.778244 |
| 2 | 2010 | 2018 | -2.0102 | -5.1570 | 2.9693 | -- | 0.307538 |
| 3 | 2018 | 2021 | -4.4400* | -7.4218 | -0.7135 | -- | 0.033193 |
| 4 | 2021 | 2024 | 2.8236 | -1.8311 | 8.8970 | -- | 0.134373 |
| *Indicates that the Annual Percentage (APC) is significantly different from zero at the alpha= 0.05 level | | | | | | | |

**Supplementary file –**

**Table 2** - **Annual Percent Change (APC) in Ectopic pregnancy admissions(per 100,000 deliveries) in England, 2005–2024.**

| Estimated Joinpoints | | | | | | | |
| --- | --- | --- | --- | --- | --- | --- | --- |
| Joinpoint | | Estimate | | Lower CI | | Upper CI | |
| 1 | | 2012 | | 2007 | | 2012 | |
| 2 | | 2015 | | 2011 | | 2018 | |
| 3 | | 2021 | | 2018 | | 2022 | |
| Annual Percentage Change | | | | | | | |
| Segment | Lower Endpoint | Upper Endpoint | APC | Lower CI | Upper CI | Test Statistic | P-Value |
| 1 | 2004 | 2012 | 2.8200* | 1.3845 | 3.8309 | -- | 0.007598 |
| 2 | 2012 | 2015 | -2.9711 | -3.9393 | 3.827 | -- | 0.15197 |
| 3 | 2015 | 2021 | 0.1836 | -1.6374 | 1.5835 | -- | 0.661868 |
| 4 | 2021 | 2024 | 4.2300* | 2.1194 | 7.1943 | -- | < 0.000001 |
| * Indicates that the Annual Percentage (APC) is significantly different from zero at the alpha= 0.05 level | | | | | | | |

**Supplementary file –**

**Methods**

2.1 Data sources

We sourced data on miscarriage and ectopic pregnancy from the Hospital Episode Statistics (HES) and the Maternity Services Data Set (MSDS) curated by NHS England. HES is a national administrative database containing detailed records of all hospital admissions, outpatient activity, and accident and emergency attendances in NHS hospitals in England. The MSDS captures information across the maternity care pathway, including additional clinical and service-level variables not available in HES.

In this study, HES and MSDS were used for distinct and complementary purposes and were not linked at the individual level. HES was used to identify hospital admissions for miscarriage and ectopic pregnancy, while MSDS was used to derive maternity-related denominators (deliveries). As these datasets capture different components of care pathways, the risk of double counting was minimised. NHS England applies standardised XML schema validation, data cleaning, and quality assurance processes, including internal consistency checks (e.g. a single booking per woman per reporting period per organisation) to minimise the double counting. (<https://digital.nhs.uk/data-and-information/publications/statistical/nhs-maternity-statistics/2024-25/data-quality-statement-msds>)

Annual counts of deliveries, miscarriages (ICD-10 codes O02.0–O02.9, O03), and ectopic pregnancies (ICD-10 O00) were extracted for the period 2004–2024. Data were stratified by Index of Multiple Deprivation (IMD) deciles (1 = most deprived, 10 = least deprived) and by maternal age groups to examine age-related distributions (2013–2024, based on data completeness). The complete IMD and age groups data were not available in the datasets prior to this time.

Deprivation was measured using the IMD, an area-level composite index derived at the level of Lower-layer Super Output Areas (~1,500 population), incorporating seven domains: income( the Income Deprivation Affecting Children Index and the Income Deprivation Affecting Older People Index ), employment, education, health, crime, barriers to housing and services, and living environment.( <https://www.gov.uk/government/statistics/english-indices-of-deprivation-2025/english-indices-of-deprivation-2025-frequently-asked-questions>)

For age groups, we divided the population into the following categories: < 20 years, 20-24 years, 25-29 years, 30-34 years, 35-39 years, 40- 44 years, 45-49 years and > 50 years.

We attempted to extract data stratified jointly by age and IMD in relation to pregnancy outcomes; however, publicly available MSDS/HES data provide counts stratified by age and deprivation decile separately, without cross-tabulated data. Analyses were therefore performed independently for age and deprivation. Individual-level record linkage was not available, precluding assessment of their independent effects.

All datasets were collated in Microsoft Excel before importing summary tables into the Joinpoint regression program (version 5.0; National Cancer Institute, USA) for analysis.

2.2 Statistical analysis

Temporal trends in miscarriage and ectopic pregnancy admissions were analysed using the joinpoint regression program (version 5.0; National Cancer Institute, USA). This method identifies statistically significant changes in trends by fitting segmented log-linear models, with each joinpoint representing a change in slope.

A log-linear model with Poisson variance was applied to annual counts, using deliveries as a proxy denominator rather than all pregnancies (including miscarriages, ectopic pregnancies, and terminations) or total conceptions, as such data are not consistently available in routine datasets. Pregnancy is typically recorded from the first antenatal booking (8–12 weeks), and early pregnancy losses may occur before this point and remain unrecorded.

The models were fitted allowing a maximum of 3 joinpoints, based on the length of the time series (20 observations). A minimum segment length of 3 observations was specified to ensure stability of estimates. The optimal number of joinpoints was selected using the Monte Carlo permutation test with 4499 permutations, in accordance with standard joinpoint methodology.

Overdispersion was present in the aggregated data which is common in large population-based datasets. While negative binomial regression would provide improved variance estimation in a traditional regression framework, it is not currently implemented within the Joinpoint modeling approach. Importantly, Poisson-based Joinpoint regression remains one of the standard methods for estimating temporal trends in surveillance research.( <https://pubmed.ncbi.nlm.nih.gov/10649300/> , <https://www.thelancet.com/journals/lanwpc/article/PIIS2666-6065(25)00318-9/fulltext>)

Model diagnostics indicated that residuals were uncorrelated, suggesting no significant autocorrelation. The Annual Percent Change (APC) quantifies the yearly change in rates, assuming a constant percentage change per year. Annual percent change (APC) and average annual percent change (AAPC) with 95% confidence intervals (CIs) were calculated. The Average Annual Percent Change (AAPC) provides a single summary measure of the trend over a fixed interval (2004–2024), calculated as a weighted average of the APCs from each segment, with weights proportional to the segment lengths.

For trend directions, "significant increase/decrease" meant a statis¬tically significant trend slope (p<0.05, based on AAPC). Non-significant trends (p>0.05) were labelled "non-statistically significant increase/ decrease" or "stable" based on AAPC values. Statistical significance was defined as p<0.05.
